# Prediction of Alzheimer’s disease biomarker status defined by the ‘ATN framework’ among cognitively healthy individuals: results from the EPAD longitudinal cohort study

**DOI:** 10.1186/s13195-020-00711-5

**Published:** 2020-11-09

**Authors:** Catherine M. Calvin, Casper de Boer, Vanessa Raymont, John Gallacher, Ivan Koychev, The European Prevention of Alzheimer’s Dementia (EPAD) Consortium

**Affiliations:** 1 Department of Psychiatry, University of Oxford, Warneford Hospital, Oxford, OX3 7JX UK; 2 Alzheimer Center Amsterdam, Amsterdam UMC, Amsterdam, Netherlands

**Keywords:** ATN framework, Risk scores, White matter lesions volume

## Abstract

**Background:**

The Amyloid/Tau/Neurodegeneration (ATN) framework has been proposed as a means of evidencing the biological state of Alzheimer’s disease (AD). Predicting ATN status in pre-dementia individuals therefore provides an important opportunity for targeted recruitment into AD interventional studies. We investigated the extent to which ATN-defined biomarker status can be predicted by known AD risk factors as well as vascular-related composite risk scores.

**Methods:**

One thousand ten cognitively healthy older adults were allocated to one of five ATN-defined biomarker categories. Multinomial logistic regression tested risk factors including age, sex, education, APOE4, family history of dementia, cognitive function, vascular risk indices (high systolic blood pressure, body mass index (BMI), high cholesterol, physical inactivity, ever smoked, blood pressure medication, diabetes, prior cardiovascular disease, atrial fibrillation and white matter lesion (WML) volume), and three vascular-related composite scores, to predict five ATN subgroups; ROC curve models estimated their added value in predicting pathology.

**Results:**

Age, APOE4, family history, BMI, MMSE and white matter lesions (WML) volume differed between ATN biomarker groups. Prediction of Alzheimer’s disease pathology (versus normal AD biomarkers) improved by 7% after adding family history, BMI, MMSE and WML to a ROC curve that included age, sex and APOE4. Risk composite scores did not add value.

**Conclusions:**

ATN-defined Alzheimer’s disease biomarker status prediction among cognitively healthy individuals is possible through a combination of constitutional and cardiovascular risk factors but established dementia composite risk scores do not appear to add value in this context.

## Background

The recognition that Alzheimer’s disease (AD) runs a prolonged preclinical course has led to a shift in interventional trials towards its pre-syndromal stages. The Amyloid/Tau/Neurodegeneration (ATN) framework [17] was proposed as means of evidencing the biological state of AD, independent of clinical manifestation. Predicting ATN status through known risk factors and derived scores may provide an important opportunity for aiding preclinical diagnosis and targeted recruitment into AD interventional studies.

Older age and the apolipoprotein *e*4 (APOE4) genotype are the leading constitutional risk factors for AD, yet modifiable vascular-related risk factors are associated with additional late-life risk of AD [24, 30, 37, 43], dementia risk [1, 12, 14, 34, 38, 41, 47] and AD pathology biomarkers in preclinical older-age individuals [31, 39, 42]. Despite continuous measures of vascular risk factors relating to ATN biomarkers, little is known about how composite risk scores for dementia relate to ATN-defined biomarker groups among cognitively healthy individuals. Such validation would support the utility of a single vascular risk score for screening high-risk individuals into disease-modifying intervention studies. However, there is no clear consensus about the optimal dementia risk score, and alternative ones have been validated according to different population samples. For example, the Cardiovascular Risk Factors, Aging and Dementia (CAIDE) risk score was originally validated by its prediction of incident dementia 20 years later [25]; the Framingham general cardiovascular score showed an association with 10-year cognitive decline [21]; and the Framingham stroke score showed significant association with contemporaneous cognitive function [9]. A recent study in middle-aged individuals compared them in relation to cognitive decline over 10 years [20] finding that all three predicted 10-year cognitive decline, with Framingham risk scores showing greater strength of association relative to CAIDE.

## Methods

### Study design and aims

Here we tested the predictive utility of validated composite risk scores in their association with ATN-defined biomarker group status in a cognitively healthy ageing sample drawn from the European Prevention of Alzheimer’s Dementia longitudinal cohort study (EPAD LCS). Our main aim was to investigate independent risk factors for AD and vascular-related composite risk scores, in relation to prediction of ATN pathology.

### Study setting

The sample is drawn from 1500 adult participants aged over 50 years from EPAD LCS (dataset V1500.0) [40, 46]. It recruited participants across 21 European sites across the full range of anticipated probability for AD development [46]. Baseline measurements included brain imaging, fluid biomarkers, cognitive performance, medical history, functional capability, physical examination and neuropsychiatric assessment. For the purposes of this preclinical AD analysis, we excluded participants with known diagnosis of dementia or Mild Cognitive Impairment (MCI) or Clinical Dementia Rating scale (CDR) consistent MCI (i.e. CDR ≥ 0.5 [15];).

### Assessments

#### Constitutional and genetic risk factors

Constitutional risk factors included age, female sex, years of education and self-reported dementia diagnosis in a first-degree relative. APOE4 genotype was determined from Taqman Genotyping of blood, analysed in a single laboratory using QuantStudio12K Flex (www.ep-ad.org).

#### Cognitive risk factors

Total Mini-Mental State Examination (MMSE) score was used to derive a measure of global cognitive function [11]. Performance scores from Repeatable Battery for the Assessment of Neuropsychological Status (RBANS) tests of episodic verbal memory, executive function and processing speed were also included as predictors. The RBANS word list learning task assesses episodic verbal memory and involves the immediate recall of 10 semantically unrelated words and is thus scored between 0 and 10; the RBANS coding task assesses executive control function, visual attention and processing speed and is scored according to the number of correct digit-coded responses during a 90-s interval.

#### Vascular risk factors

We included the continuous variables of body mass index (BMI), systolic BP and binary medical history variables: hypertensive medication use, past or current diabetes, hypercholesterolaemia and cardiovascular disease (CVD; myocardial infarction, angina pectoris, coronary insufficiency, intermittent claudication, congestive heart failure, arrythmia). Self-reported smoking was included as a binary variable (‘ever’ versus ‘never’). Physical inactivity was binary coded according to self-reported frequency of < 2 weekly sessions of moderate or vigorous physical activity. The proportion of white matter lesion (WML) volume per whole brain volume was derived from volumetric MRI data—the brain imaging protocol is described elsewhere [40].

#### Composite risk scores

Specific risk factors were used to derive three alternative composite scores:

##### CAIDE risk score

The original scoring system for CAIDE [25] uses information on age, sex, education, high BP, BMI, total cholesterol and physical inactivity. We allocated a score of 2 to participants who self-reported hypercholesterolaemia (instead of CAIDE’s criterion of > 6.5 mmol/L total cholesterol) in the absence of blood biomarker data in EPAD LCS.

##### Framingham general cardiovascular disease risk score

The sex-adjusted scoring system [6] uses information on age, systolic BP, hypertensive medication, diabetes, total and HDL cholesterol and smoking. In the absence of blood biomarker data for scoring total and HDL cholesterol, we applied the scoring system for self-reported diabetes to self-reported hypercholesterolaemia.

##### Framingham stroke risk score

The sex-adjusted scoring system for the Framingham stroke composite score [7] uses information on age, systolic BP, hypertensive medication, diabetes, smoking, prior CVD, atrial fibrillation and ventricular hypertrophy.

#### CSF biomarkers

Cerebral spinal fluid (CSF) samples were measured for amyloid-beta 42 (Aß42) and pathologic tau (p-Tau) in a single laboratory using the Roche Elecsys System. The cut-off for definition of normal Aß42 pathology (A+) was < 1025 pg/ml, derived by the present authors using Gaussian Mixture Modeling. This approach is based upon previous work in which the assay used in EPAD was validated in two independent cohorts [13]. According to this method, cut-offs are defined either by the intersection point of the two components or by the mean ± standard deviations. A similar method was used to define the cut-off of > 24 pg/ml for p-Tau pathology (T+).

#### Neurodegeneration biomarker

Scheltens’ visual rating scale [44] for medial temporal lobe atrophy based upon structural MRI images was used as the neurodegenerative marker. Neurodegenerative pathology (N+) was defined using decade-specific cut-off values to optimise sensitivity and specificity [5]: scores > 1 f participants less than 65 years old, > 1.5 for 65 to 74-year-olds, and, and > 2 for participants older than 75 years.

#### ATN-defined biological categories

Participants were categorised into five subgroups as characterised in the ATN Framework [17]:

(i) *Normal AD biomarkers*: A−T−(N)−
(ii) *Alzheimer’s pathologic change*: A+T−(N)−
(iii) *Alzheimer’s disease*: A+T+(N)±
(iv) *Alzheimer’s and concomitant non-Alzheimer’s pathologic change*: A+T−(N)+
(v) *Non-AD pathologic change*: A−T ± (N)+; A−T+(N)−

### Statistical analysis

Due to multiple testing throughout, we set significance levels using Bonferroni correction.

Odds ratios and 95% confidence intervals were estimated in age- and sex-adjusted logistic regression models to observe the extent to which individual risk factors related to brain pathology measured according to A+, T+ and N+, respectively. Following this, comparisons of risk factors among the five ATN-defined biomarker groups were formally tested using one-way ANOVA or chi-squared tests for continuous and categorical binary variables, respectively. When overall group differences were observed, we investigated which specific group comparisons contributed most to these, by estimating chi-square residuals for categorical binary variables, and conducting Bonferroni tests for continuous variables.

To adjust for the potential confounding effects of age and sex on observed group differences, we conducted multinomial logistic regression models, including age, sex and study site each time, to predict ATN biomarker group membership according to each risk factor. The normal AD biomarker group was the reference category. To understand whether associations had sex or genetic-risk specific effects, we reran the models to include interaction terms between each risk factor with age and APOE4 genotype, respectively.

Finally, we constructed receiver operating characteristic (ROC) curves and estimated areas under the curve (AUC) for models that included the significant risk factors from the previous multinomial logistic regression and each of the composite risk scores, respectively. A basic model (1) included age, sex and APOE4, and subsequent models included:

i) basic model + each significant risk factor in turn;
ii) basic model + all significant risk factors; and
iii) basic model + each of three composite risk scores.

Differences from the basic model were evaluated with the chi-square test.

## Results

Among 1500 participants, 82 individuals had a clinical diagnosis of dementia or MCI. An additional 171 participants were excluded due to CDR score ≥ 0.5. A further 237 participants had missing data on one or more ATN biomarkers, and their exclusion led to an analytic sample of 1010 individuals (mean age 64.6 ± 6.8 years; 59% female). Amyloid-beta pathology (A+) was observed in 309 (30.6) participants, pathologic tau pathology (T+) in 158 (15.6%) and neurodegenerative pathology in 90 (8.9%) participants. ATN-defined criteria classed 56.1% (*n* = 567) as ‘normal AD biomarkers’, 20.9% (*n* = 211) as ‘Alzheimer’s pathologic change’, 6.6% (*n* = 67) as ‘Alzheimer’s disease’, 3.1% (*n* = 31) as ‘Alzheimer’s and concomitant non-Alzheimer’s pathologic change’ and 13.3% (*n* = 134) as ‘non-AD pathologic change’.

### Risk factors associated with pathology groups A+, T+ and N+

**Fig. 1:**
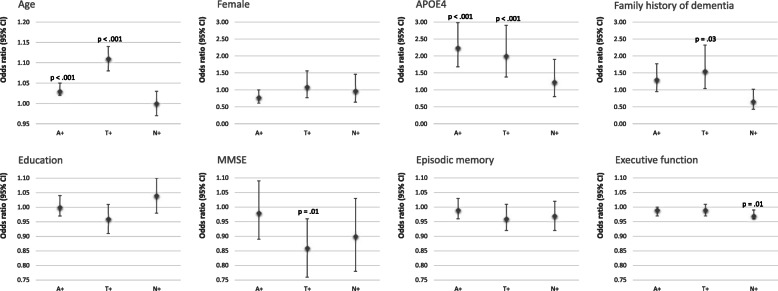
Age- and sex-adjusted odds ratios and 95% confidence intervals for the associations of constitutional- and cognitive-risk factors and amyloid-beta pathology (A+), p-Tau pathology (T+) and neurodegeneration (N+), respectively. Statistically significant *P* values are indicated (*p* < 0.05)

**Fig. 2:**
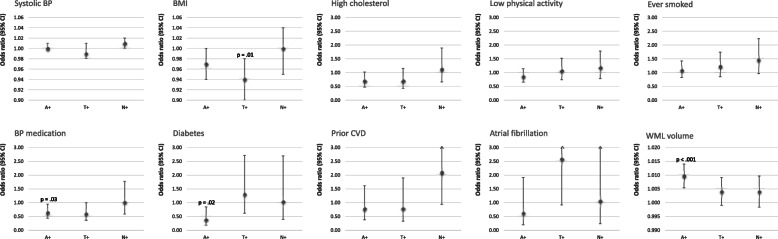
Age- and sex-adjusted odds ratios and 95% confidence intervals for the associations of vascular risk factors and amyloid-beta pathology (A+), p-Tau pathology (t+) and neurodegeneration (N+), respectively. Statistically significant *P* values are indicated (*p* < 0.05)

Individual risk factors were first tested for their respective associations with A+, T+ and N+, respectively, in logistic regression models that adjusted for age and sex (see Fig. 1 for odds ratios according to constitutional and cognitive risk factors; Fig. 2 for effects according to vascular risk factors.) In terms of constitutional risk factors, we found that age and APOE4 carriership both increased the risk of amyloid (OR = 1.03, 95% CI 1.02–1.05, *p* < 0.001; OR = 2.24, 95% CI 1.68–2.98, *p* < 0.001) and tau pathology, respectively (OR = 1.11, 95%CI 1.08–1.14, *p* < 0.001; OR = 2.00, 95% CI 1.38–2.90, *p* < 0.001), while the family history of dementia associated with tau positivity only (OR = 1.55, 95%CI 1.04–2.32, *p* = 0.03). Lower global cognition scores (MMSE) associated with tau positivity (OR = 0.86, 95% CI 0.76–0.96, *p* = 0.01) while lower executive function scores associated with neurodegeneration (OR = 0.97, 95% CI 0.96–0.99, *p* = 0.01). In terms of cardiovascular risk factors, we found evidence for lower BMI associating with tau pathology (OR = 0.94, 95% CI 0.90–0.98, *p* = 0.01) whereas amyloid pathology was linked to lower use of hypertensive therapy, lack of diabetes diagnosis and increased WML volumes relative to no pathology (OR = 0.64, 95% CI 0.43–0.95, *p* = 0.03; OR = 0.39, 95% CI 0.18–0.85, *p* = 0.02; OR = 1.010, 95% CI 1.005–1.014, *p* < 0.001).

### ATN group differences on risk factors

Table 1 provides a comparison of the ATN pathology groups according to descriptive statistics of each risk factor and CAIDE and Framingham risk scores. The groups differed on mean age, BMI, WML, MMSE and episodic memory and executive function scores, and on the frequency of APOE4 carrier status and family history. Further analyses clarified the specific group comparisons responsible for these differences (Table 2). The main observations were as follows: the group with Alzheimer’s disease biomarkers (versus normal AD biomarkers) were on average older, with higher WML volume, lower cognitive scores, higher Framingham stroke scores, and a higher frequency of APOE4 carriers; the Alzheimer’s pathologic change group was similar to the normal AD biomarker group in respect to mean age and cognition, and family history frequency, but showed elevated WML volume and a higher frequency of APOE4 carriers that was more consistent with AD pathology. The non-AD pathology group was notable for having the lowest frequency of family history and for a mean WML volume akin to the group with normal AD biomarkers, but with higher mean age and Framingham stroke scores, similar to the Alzheimer’s biomarker group.

**Table 1:** Descriptive data on AD risk factors according to ATN subgroups

	Total	Normal AD biomarkers	Alzheimer’s pathologic change	Alzheimer’s disease	Alzheimer’s and non-AD pathologic change	Non-AD pathologic change	***P*** value*
*N*	1010	567	211	67	31	134	
Risk exposures							
Age, years	64.6 ± 6.8	63.6 ± 6.6	64.7 ± 7.1	69.1 ± 5.9	62.4 ± 7.1	66.7 ± 6.2	**< 0.001**
Sex, female	58.6%	60.1%	54.0%	56.7%	51.6%	60.5%	0.51
Education, years	14.7 ± 3.7	14.8 ± 3.7	14.9 ± 3.7	13.9 ± 3.8	15.8 ± 3.5	14.5 ± 3.6	0.14
APOE4	37.5%	32.2%	47.5%	69.2%	36.7%	34.9%	**< 0.001**
Family history	68.5%	72.1%	69.2%	86.6%	61.3%	61.2%	**0.003**
Systolic BP	134.8 ± 17.8	133.5 ± 17.6	136.4 ± 17.9	137.8 ± 17.0	136.9 ± 18.1	136.0 ± 18.8	0.10
BMI	26.4 ± 4.3	26.4 ± 4.3	26.5 ± 4.3	24.8 ± 3.7	27.2 ± 4.5	26.2 ± 4.6	0.03
High cholesterol	16.3%	17.3%	14.7%	11.9%	9.7%	17.2%	0.59
Physical inactivity	43.0%	43.3%	38.3%	43.3%	36.7%	45.9%	0.61
Ever smoked	54.2%	52.1%	53.1%	58.2%	60.0%	59.4%	0.50
BP medication	15.5%	17.6%	13.3%	13.4%	16.1%	15.7%	0.63
Diabetes	4.5%	4.9%	1.9%	6.0%	0.0%	8.2%	0.05
Prior CVD	4.1%	3.7%	2.8%	4.5%	6.5%	5.2%	0.75
Atrial fibrillation	1.9%	1.6%	1.4%	1.5%	0.0%	3.7%	0.44
WML volume*	12.9 ±	9.3 ± 26.2	17.3 ± 35.2	27.8 ± 48.5	24.1 ± 42.8	11.2 ± 27.8	**< 0.001**
MMSE	28.8 ± 1.3	28.9 ± 1.3	28.9 ± 1.2	28.2 ± 1.9	29.0 ± 1.3	28.6 ± 1.2	**< 0.001**
Episodic verbal memory	29.1 ± 4.2	29.4 ± 4.2	29.0 ± 4.2	27.4 ± 4.5	29.7 ± 3.6	28.5 ± 4.3	**0.002**
Executive function	46.3 ± 10.3	47.5 ± 9.9	45.5 ± 10.4	41.6 ± 9.5	47.5 ± 11.8	44.3 ± 11.2	**< 0.001**
Composite scores							
CAIDE	6.22 ± 1.83	6.16 ± 1.80	6.19 ± 1.87	6.48 ± 2.00	6.00 ± 1.60	6.42 ± 1.87	0.43
Framingham CVD	14.0 ± 3.6	13.7 ± 3.6	13.8 ± 3.7	15.3 ± 3.3	13.2 ± 3.3	14.7 ± 3.3	**< 0.001**
Framingham stroke	9.32 ± 4.04	8.82 ± 3.98	9.33 ± 3.97	11.13 ± 3.66	8.97 ± 3.35	10.55 ± 4.25	**< 0.001**

Data points represent means ± SDs for continuous variables and percentages for categorical binary variables

*P values are significance levels for one-way ANOVA or chi-squared tests assessing group differences on continuous and categorical binary variables respectively; those in bold survive Bonferroni correction (0.5/21 = 0.024)

Abbreviations: *A* ß-amyloid pathology, *T* P-Tau pathology, *N* neurodegenerative pathology

**Table 2:** ATN group differences by risk factor

		Normal AD biomarkers	Alzheimer’s pathologic change	Alzheimer’s disease	Alzheimer’s and non-AD pathologic change	Non-AD pathology
Bonferroni test for subgroup comparisons						
Risk factors (continuous)		*P* value	*P* value	*P* value	*P* value	
Age	Alzheimer’s pathologic change	1.0				
	Alzheimer’s disease	**< 0.001**	**< 0.001**			
	AD/non-AD pathologic change	1.0	0.82	**< 0.001**		
	Non-AD pathology	**< 0.001**	0.07	0.15	0.02	–
WML volume*	Alzheimer’s pathologic change	0.02				
	Alzheimer’s disease	**< 0.001**	0.14			
	AD/non-AD pathologic change	0.10	1.0	1.0		
	Non-AD pathology	1.0	0.72	**0.003**	0.33	–
Framingham CVD	Alzheimer’s pathologic change	1.0				
	Alzheimer’s disease	0.02	0.03			
	AD /non-AD pathologic change	1.0	1.0	0.07		
	Non-AD pathology	0.08	0.18	1.0	0.30	–
Framingham stroke	Alzheimer’s pathologic change	1.0				
	Alzheimer’s disease	**< 0.001**	0.01			
	AD/non-AD pathologic change	1.0	1.0	0.13		
	Non-AD pathology	**< 0.001**	0.06	1.0	0.49	–
MMSE	Alzheimer’s pathologic change	1.0				
	Alzheimer’s disease	**0.005**	**0.003**			
	AD/non-AD pathologic change	1.0	1.0	0.08		
	Non-AD pathology	1.0	0.73	0.35	1.0	–
Verbal learning	Alzheimer’s pathologic change	1.0				
	Alzheimer’s disease	0.02	0.08			
	AD/non-AD pathologic change	1.0	1.0	0.13		
	Non-AD pathology	1.0	1.0	1.0	1.0	–
Coding	Alzheimer’s pathologic change	1.0				
	Alzheimer’s disease	**< 0.001**	0.09			
	AD/non-AD pathologic change	1.0	1.0	0.09		
	Non-AD pathology	0.12	1.0	0.84	1.0	–
Chi-square residuals test for evaluating subgroup contribution to group differences						
Risk factors (binary*)*		Adjusted *R*	Adjusted *R*	Adjusted *R*	Adjusted *R*	Adjusted *R*
APOE4		**− 4.74**	**3.18**	**5.45**	− 0.10	− 0.65
Family history		− 0.22	0.25	**3.28**	− 0.87	− 1.92

*P* values in bold survive Bonferroni correction (0.5/56 = 0.009). Adjusted standardised residuals (*R*) in bold represent significant subgroup effects, i.e. *R* > 2 indicates that the number of cases with a risk factor within the ATN subgroup is significantly larger than expected if the null hypothesis is true (*p* < 0.05); *R* < −2 indicates that the numbers of cases with a risk factor within the ATN subgroup is significantly smaller than expected if the null hypothesis is true

### Predicting ATN group membership in age- and sex-adjusted models

To adjust for the confounding effects of age and sex prediction of ATN group membership by risk factors were tested in age- and sex-adjusted multinomial logistic regression models using the normal AD biomarker group as the reference (Table 3). Age was significantly greater in the AD (RR = 1.14, 95%CI 1.09–1.19, *p* < 0.001) and non-AD pathology groups (RR = 1.07, 95%CI 1.04–1.10, *p* < 0.001), but not in Alzheimer’s pathologic change (RR = 1.02, 95%CI 1.00–1.05, *p* = 0.08) or combined AD and non-AD pathologic change (RR = 0.97, 95%CI 0.92–1.02, *p* = 0.25). APOE4 carriership was predictive of membership to both AD biomarker groups (AD pathologic change: RR = 1.93, 95%CI 1.37–2.72, *p* < 0.001; AD pathology: RR 6.48, 95%CI 3.57–11.8, *p* < 0.001) but was not significantly different in the non-AD pathology group (RR = 1.27, 95%CI 0.83–1.93, *p* = 0.27), and AD/non-AD pathologic change group (RR = 1.09, 95%CI 0.50–2.36, *p* = 0.82) relative to the normal AD biomarker group. Family history was predictive of Alzheimer’s pathology (RR = 4.12, 95%CI 1.93–8.77, *p* < 0.001) but not Alzheimer’s pathologic change (RR 1.00, 95%CI 0.70–1.44, *p* = 0.99), AD/non-AD pathologic change (RR = 0.63, 95%CI 0.29–1.37, *p* = 0.25) or non-AD pathology (RR = 0.75, 95%CI 0.50–1.14, *p* = 0.18). Among the vascular risk factors, the effect of WML volume survived adjustment for age and sex. WML volume was positively associated with Alzheimer’s pathologic change (RR = 1.008, 95% CI 1.003–1.014, *p* = 0.002), Alzheimer’s pathology (RR = 1.014, 95% CI 1.007–1.020, *p* < 0.001) and AD/non-AD pathologic change (RR = 1.012; 95%CI 1.004–1.021, *p* = 0.005). Furthermore, after adjusting for age and sex lower BMI was associated with the Alzheimer’s pathology group (RR = 0.88, 95% CI 0.82–0.95, *p* = 0.001). There were no vascular risk factors that significantly differentiated non-AD pathology relative to normal AD biomarkers. The group differences in MMSE and RBANS tests were no longer evident after adjustment for age and sex, with the exception that lower MMSE score was associated with AD pathology (RR = 0.79, 95%CI − 0.68–0.93, *p* = 0.006).

**Table 3:** Age- and sex-adjusted effects of risk factors on ATN subgroups: multinomial logistic regression

		Predicting Alzheimer’s pathologic change		Predicting Alzheimer’s disease		Predicting AD/non-AD pathologic change		Predicting non-AD pathology	
	*N*	RRR (95% CI)	*P*	RRR (95% CI)	*P*	RRR (95% CI)	*P*	RRR (95% CI)	*P*
Risk exposures									
1. Age, years	1010	1.02 (1.00, 1.05)	0.08	1.14 (1.09, 1.19)	**1.3 × 10**^**−09**^	0.97 (0.92, 1.02)	0.25	1.07 (1.04, 1.10)	**2.9 × 10**^**−06**^
2. Sex, female	1010	0.80 (0.58, 1.10)	0.17	0.93 (0.55, 1.56)	0.78	0.71 (0.34, 1.47)	0.36	1.05 (0.71, 1.56)	0.79
3. Education, years	1010	1.00 (0.96, 1.05)	0.87	0.95 (0.89, 1.02)	0.18	1.07 (0.97, 1.18)	0.16	0.99 (0.94, 1.04)	0.75
4. APOE4	955	1.93 (1.37, 2.72)	**1.6 × 10**^**−04**^	6.48 (3.57, 11.8)	**8.5 × 10**^**−10**^	1.09 (0.50, 2.36)	0.82	1.27 (0.83, 1.93)	0.27
5. Family history	1010	1.00 (0.70, 1.44)	0.99	4.12 (1.93, 8.77)	**2.5 × 10**^**−04**^	0.63 (0.29, 1.37)	0.25	0.75 (0.50, 1.14)	0.18
6. Systolic BP	1009	1.00 (1.00, 1.01)	0.35	1.00 (0.98, 1.02)	1.0	1.01 (0.99, 1.03)	0.32	1.00 (0.99, 1.01)	0.95
7. BMI	1006	0.98 (0.94, 1.02)	0.29	0.88 (0.82, 0.95)	**0.001**	1.02 (0.94, 1.10)	0.65	0.98 (0.93, 1.02)	0.30
8. High cholesterol	1010	0.75 (0.48, 1.18)	0.21	0.49 (0.22, 1.08)	0.08	0.52 (0.15, 1.77)	0.30	0.84 (0.51, 1.40)	0.51
9. Physical inactivity	1005	0.86 (0.62, 1.19)	0.36	1.01 (0.59, 1.71)	0.98	0.83 (0.38, 1.79)	0.64	1.13 (0.77, 1.67)	0.53
10. Ever smoked	1005	1.09 (0.79, 1.50)	0.62	1.34 (0.79, 2.28)	0.28	1.54 (0.72, 3.29)	0.27	1.39 (0.94, 2.05)	0.10
11. BP medication	1010	0.61 (0.38, 0.97)	0.04	0.48 (0.23, 1.04)	0.06	0.87 (0.32, 2.40)	0.79	0.69 (0.41, 1.18)	0.18
12. Diabetes	1010	0.32 (0.11, 0.94)	0.04	0.81 (0.26, 2.51)	0.72	–		1.44 (0.68, 3.05)	0.34
13. Prior CVD	1010	0.68 (0.27, 1.73)	0.42	0.71 (0.19, 2.57)	0.60	2.00 (0.43, 9.23)	0.38	1.10 (0.45, 2.70)	0.84
14. Atrial fibrillation	1010	0.87 (0.23, 3.27)	0.84	0.81 (0.10, 6.73)	0.84	–		2.22 (0.72, 6.86)	0.17
15. WML volume*	988	1.008 (1.003, 1.014)	**0.002**	1.014 (1.007, 1.020)	**4.0 × 10**^**−05**^	1.012 (1.004, 1.021)	0.**005**	1.002 (0.995, 1.010)	0.52
16. MMSE	1009	1.02 (0.90, 1.16)	0.74	0.79 (0.68, 0.93)	**0.006**	1.04 (0.77, 1.41)	0.80	0.91 (0.79, 1.05)	0.18
17. Episodic verbal memory	1007	1.00 (0.96, 1.04)	0.95	0.95 (0.89, 1.01)	0.10	1.03 (0.94, 1.14)	0.53	0.98 (0.93, 1.03)	0.38
18. Executive function	1007	0.98 (0.97, 1.00)	0.05	0.97 (0.95, 1.00)	0.02	0.99 (0.96, 1.03)	0.70	0.98 (0.96, 1.00)	0.08
Composite scores									
19. CAIDE	1002	0.92 (0.77, 1.10)	0.34	1.00 (0.75, 1.32)	0.98	0.86 (0.56, 1.32)	0.49	1.05 (0.85, 1.29)	0.65
20. Framingham CVD	1005	0.90 (0.70, 1.12)	0.33	0.84 (0.57, 1.23)	0.37	0.96 (0.56, 1.64)	0.88	0.97 (0.74, 1.27)	0.82
21. Framingham stroke	1005	0.99 (0.78, 1.27)	0.96	0.94 (0.65, 1.37)	0.75	1.31 (0.77, 2.24)	0.32	1.23 (0.93, 1.61)	0.14

All models additionally adjust for study site (*n* = 21). Effects sizes are relative risk ratios (RRR) and their 95% confidence intervals. For the full sample (*n* = 1010) the groups sizes are as follows: normal AD biomarkers: *n* = 567; Alzheimer’s pathologic change: *n* = 211; AD: *n* = 67; AD and non-AD pathologic change: *n* = 31; non-AD pathologic change: *n* = 134. Composite scores are entered into models as *z*-scores. *P* values in bold survive Bonferroni correction (0.5/84 = 0.006)

*Proportion of total brain volume. No significant interaction terms were observed between individual vascular risk factors and age nor sex, respectively, except that sex and MMSE score in predicting Alzheimer’s pathologic change (*p* = 0.006), and age and APOE4 in predicting Alzheimer’s pathologic change (*p* < 0.001)

Multinomial logistic regression models to predict ATN group membership were then tested according to CAIDE and Framingham composite risk scores. On adjustment for age and sex effects, the different composite scores were not predictive of ATN group membership.

### Potential interactions between APOE4 and risk factors in predicting ATN

We repeated the above multinomial logistic regression models with an additional interaction term for each risk factor and APOE4 carriership. The only significant interaction term was between age and APOE4 status, which was significant in predicting Alzheimer’s pathologic change versus normal AD biomarkers (*p* < 0.001). A closer examination of this effect showed that APOE4 carriers were younger than non-carriers in participants with normal AD biomarkers (61.8 ± 6.1 vs 65.0 ± 6.7 years), while APOE4 carriers were older than non-carriers among those with Alzheimer’s pathologic change (65.2 ± 6.5 vs 64.1 ± 7.8 years). We therefore included an interaction term for age and APOE4 in subsequent models of Alzheimer’s pathologic change.

### ROC models for predicting ATN subgroup pathology versus no pathology

For the prediction of each ATN pathology group relative to no pathology, the following logistic regression models were run: model 1: age, sex and APOE4 status (including an age X APOE4 interaction term for Alzheimer’s pathologic change); model 2: model 1 plus family history; model 3: model 1 plus BMI; model 4: model 1 plus WML volume; model 5: model 1 plus MMSE; model 6: all preceding risk factors; and model 7a, b and c: model 1 plus each composite risk score (Table 4).

**Table 4:** ROC curve results of predicting ATN biomarker groups according to individual and combined risk factors

	Basic model (model 1)	Model 1 + family history	Model 1 + BMI	Model 1 + WML volume	Model 1 + MMSE	Model 1 + all previous risk factors	Model 1 + CAIDE score	Model 1 + Framingham CVD score	Model 1 + Framingham stroke score
Alzheimer’s pathologic change									
Age, years	0.99 (0.96, 1.03)	0.99 (0.96, 1.03)	1.00 (0.96, 1.03)	0.99 (0.96, 1.03)	0.99 (0.96, 1.03)	0.99 (0.96, 1.02)	1.00 (0.97, 1.03)	1.00 (0.96, 1.05)	1.00 (0.96, 1.04)
Sex, female	0.81 (0.57, 1.15)	0.83 (0.58, 1.17)	0.79 (0.55, 1.12)	0.86 (0.61, 1.22)	0.82 (0.65, 1.03)	0.81 (0.64, 1.02)	0.75 (0.52, 1.09)	0.86 (0.59, 1.27)	0.81 (0.57, 1.15)
APOE4*	0.00 (0.00, 0.07)	0.00 (0.00, 0.07)	0.00 (0.00, 0.08)	0.00 (0.00, 0.08)	0.00 (0.00, 0.08)	0.00 (0.00, 0.09)	0.00 (0.00, 0.08)	0.00 (0.00, 0.08)	0.00 (0.00, 0.07)
Family history		0.83 (0.56, 1.22)				0.88 (0.59, 1.32)			
BMI			0.98 (0.94, 1.02)			0.98 (0.94, 1.03)			
WML volume^†^				1.01 (1.01, 1.01)		1.01 (1.00, 1.01)			
MMSE score*					0.00 (0.00, 0.12)	0.00 (0.00, 0.21)			
Composite risk score							0.89 (0.72, 1.09)	0.91 (0.71, 1.17)	0.98 (0.76, 1.27)
**AUC (95% CI)**	0.63 (0.58, 0.68)	0.64 (0.59, 0.69)	0.63 (0.58, 0.67)	0.66 (0.61, 0.71)	0.65 (0.60, 0.69)	0.66 (0.61, 0.71)	0.63 (0.58, 0.68)	0.63 (0.58, 0.68)	0.63 (0.58, 0.68)
***P*** **value**	–	0.33	0.70	0.02	0.25	0.09	0.84	0.95	0.68
Alzheimer’s disease									
Age, years	1.19 (1.13, 1.25)	1.21 (1.15, 1.28)	1.18 (1.13, 1.24)	1.18 (1.12, 1.24)	1.18 (1.12, 1.24)	1.19 (1.13, 1.26)	1.19 (1.13, 1.25)	1.21 (1.33, 1.30)	1.21 (1.14, 1.30)
Sex, female	1.03 (0.57, 1.84)	0.89 (0.49, 1.62)	0.84 (0.46, 1.55)	1.14 (0.62, 2.07)	0.89 (0.49, 1.63)	0.63 (0.32, 1.25)	0.94 (0.50, 1.76)	1.00 (0.56, 1.80)	1.20 (0.63, 2.30)
APOE4	7.89 (4.18, 14.9)	6.96 (3.68, 13.2)	7.78 (4.11, 14.8)	8.91 (4.59, 17.3)	7.94 (4.19, 15.1)	7.87 (3.96, 15.6)	8.14 (4.28, 15.5)	8.09 (4.27, 15.3)	8.15 (4.30, 15.5)
Family history		4.08 (1.70, 9.81)				4.63 (1.79, 12.0)			
BMI			0.88 (0.81, 0.96)			0.85 (0.76, 0.94)			
†WML volume				1.01 (1.00, 1.02)		1.02 (1.01, 1.03)			
MMSE score					0.78 (0.66, 0.92)	0.78 (0.65, 0.94)			
Composite risk score							0.89 (0.64, 1.22)	0.82 (0.53, 1.26)	0.78 (0.51, 1.21)
**AUC (95% CI)**	0.82 (0.77, 0.88)	0.84 (0.79, 0.89)	0.84 (0.78, 0.89)	0.84 (0.78, 0.90)	0.84 (0.79, 0.89)	0.89 (0.85, 0.93)	0.82 (0.77, 0.88)	0.82 (0.77, 0.88)	0.83 (0.77, 0.88)
***P*** **value**	–	0.15	0.18	0.07	0.11	**0.0002**	0.80	0.80	0.45
AD and non-AD pathologic change									
Age, years	0.98 (0.92, 1.04)	0.97 (0.91, 1.03)	0.98 (0.92, 1.04)	0.98 (0.91, 1.04)	0.98 (0.92, 1.04)	0.97 (0.91, 1.03)	0.98 (0.92, 1.04)	0.97 (0.90, 1.06)	0.95 (0.87, 1.03)
Sex, female	0.72 (0.34, 1.52)	0.78 (0.36, 1.66)	0.75 (0.35, 1.60)	0.78 (0.37, 1.68)	0.72 (0.34, 1.54)	0.89 (0.41, 1.93)	0.68 (0.31, 1.49)	0.71 (0.31, 1.60)	0.71 (0.34, 1.52)
APOE4	0.99 (0.44, 2.22)	1.06 (0.47, 2.39)	0.98 (0.44, 2.21)	1.11 (0.49, 2.53)	0.99 (0.44, 2.22)	1.15 (0.50, 2.64)	1.02 (0.45, 2.32)	0.98 (0.43, 2.23)	0.95 (0.42, 2.15)
Family history		0.49 (0.22, 1.09)				0.54 (0.24, 1.23)			
BMI			1.05 (0.96, 1.14)			1.05 (0.96, 1.15)			
WML volume^†^				1.01 (1.00, 1.02)		1.01 (1.00, 1.02)			
MMSE score					1.02 (0.75, 1.38)	1.01 (0.74, 1.37)			
Composite risk score							0.90 (0.57, 1.42)	1.03 (0.60, 1.75)	1.35 (0.80, 2.27)
**AUC (95% CI)**	0.56 (0.44, 0.68)	0.62 (0.50, 0.72)	0.57 (0.45, 0.68)	0.63 (0.51, 0.75)	0.56 (0.44, 0.68)	0.68 (0.56, 0.80)	0.56 (0.45, 0.68)	0.56 (0.44, 0.68)	0.58 (0.47, 0.69)
***P*** **value**	–	0.33	0.84	0.11	0.92	0.06	0.96	0.88	0.45
Non-AD pathology									
Age, years	1.07 (1.04, 1.11)	1.07 (1.03, 1.10)	1.07 (1.04, 1.11)	1.07 (1.04, 1.11)	1.07 (1.03, 1.10)	1.06 (1.03, 1.10)	1.07 (1.03, 1.11)	1.05 (1.00, 1.09)	1.07 (1.03, 1.12)
Sex, female	1.11 (0.74, 1.68)	1.14 (0.75, 1.72)	1.10 (0.73, 1.66)	1.13 (0.75, 1.71)	1.08 (0.71, 1.63)	1.09 (0.71, 1.66)	1.19 (0.77, 1.84)	1.14 (0.75, 1.72)	1.11 (0.71, 1.74)
APOE4	1.38 (0.90, 2.13)	1.43 (0.92, 2.21)	1.39 (0.90, 2.14)	1.39 (0.90, 2.15)	1.38 (0.89, 2.12)	1.44 (0.93, 2.23)	1.35 (0.87, 2.09)	1.35 (0.87, 2.08)	1.38 (0.89, 2.13)
Family history		0.68 (0.44, 1.04)				0.68 (0.44, 1.04)			
BMI			0.99 (0.94, 1.04)			0.98 (0.93, 1.03)			
WML volume^†^				1.00 (1.00, 1.01)		1.00 (1.00, 1.01)			
MMSE score					0.89 (0.77, 1.03)	0.88 (0.76, 1.02)			
Composite risk score							1.10 (0.88, 1.37)	1.25 (0.95, 1.64)	1.00 (0.76, 1.33)
**AUC (95% CI)**	0.63 (0.58, 0.68)	0.64 (0.59, 0.69)	0.63 (0.58, 0.69)	0.63 (0.58, 0.68)	0.64 (0.59, 0.69)	0.66 (0.60, 0.71)	0.63 (0.58, 0.68)	0.63 (0.58, 0.69)	0.63 (0.58, 0.68)
***P*** **value**	–	0.32	0.31	0.52	0.37	0.08	0.92	0.55	0.78

All models estimate the discriminative accuracy of predicting ATN-defined biomarker group versus the normal AD biomarker group

*Models predicting AD pathologic change include age by APOE4 interaction term and sex by MMSE interaction. *P* values in bold survive Bonferroni correction (0.5/32 = 0.016)

^†^Proportion of total brain volume. Group sizes: *n* = 525 participants with normal AD biomarkers versus: Alzheimer’s pathologic change (*n* = 187); Alzheimer’s disease (*n* = 64); AD and non-AD pathologic change (*n* = 29); non-AD pathology (*n* = 122). *P* values in bold survive Bonferroni correction (0.5/32 = 0.016)

**Fig. 3:**
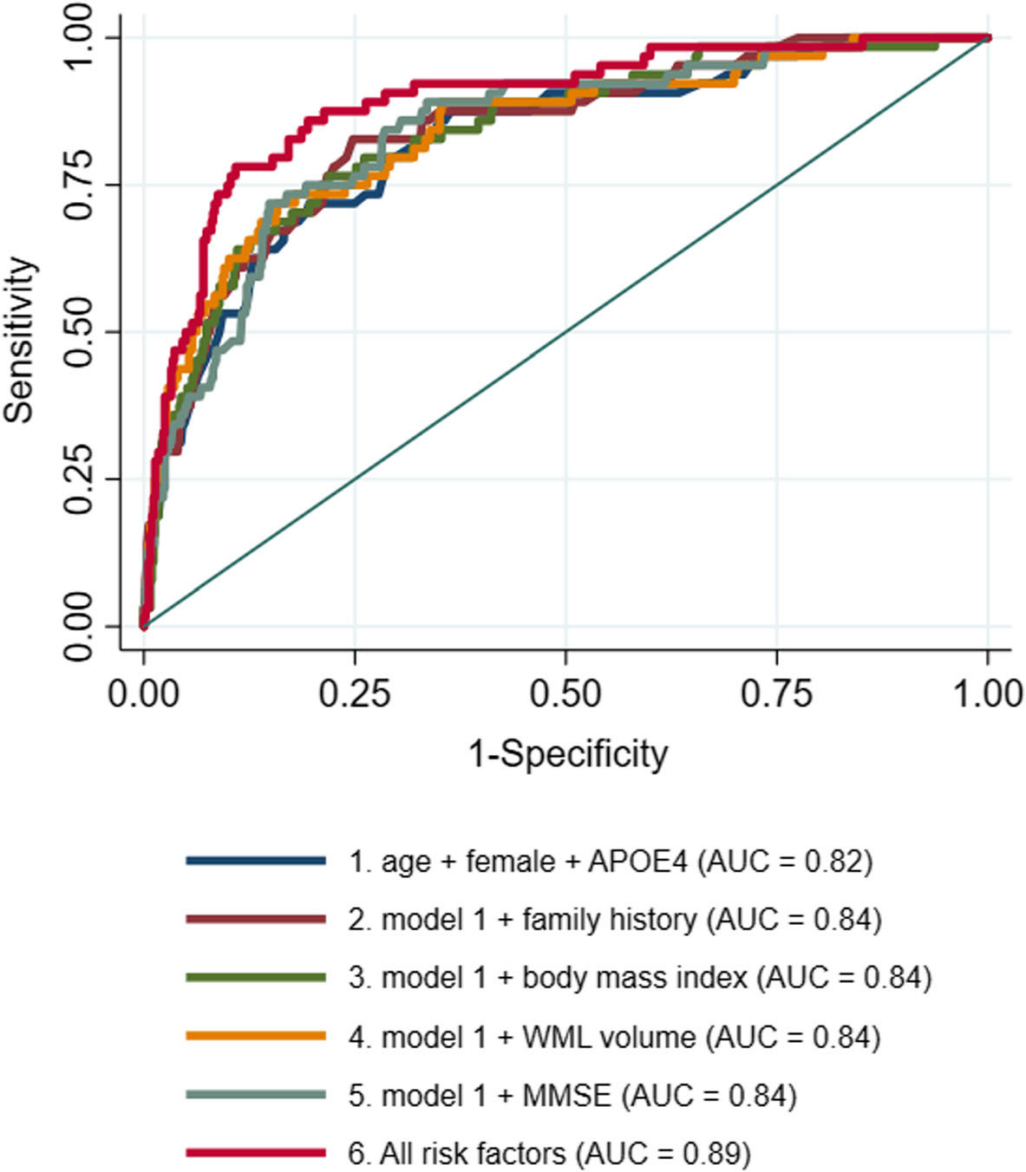
ROC curve showing improvement to the discriminative accuracy of predicting Alzheimer’s disease vs normal AD biomarkers by cumulative risk factors

In the basic model, AUC was greatest for predicting AD pathology (AUC = 0.82, 95%CI 0.77–0.88), followed by Alzheimer’s pathologic change (AUC = 0.63, 95%CI 0.58–0.68) and non-AD pathology (AUC = 0.63, 95%CI 0.58–0.68) and then AD and non-AD pathologic change (AUC = 0.56, 95%CI 0.44–0.68). The significant effect of family history in model 2 for predicting Alzheimer’s pathology did not lead to a more predictive model than model 1 (AUC = 0.84, 95%CI 0.79 to 0.89), as it may have displaced some of the APOE4 effects. Further inclusion of individual risk factors to model 1 did not improve its prediction of Alzheimer’s pathology. However, in model 6, combining family history, BMI, MMSE and WML volume with basic model risk factors significantly improved the AD pathology AUC (AUC = 0.89, 95%CI 0.85–0.93, *p* < 0.001; Fig. 3). Only the addition of WML to the basic model seemed to improve the prediction of Alzheimer’s pathologic change (AUC = 0.66, 95%CI 0.61 to 0.71, *p* = 0.02), although this did not survive Bonferroni correction. There were no significant improvements observed from the basic model in predicting AD and non-AD pathologic change and non-AD pathology, respectively, versus no pathology. Addition of the three composite risk scores made no improvement over the basic models for any pathology group.

## Discussion

In this analysis, we found that among older cognitively healthy adults, Alzheimer’s disease pathology and Alzheimer’s pathologic change defined by the ATN Framework are predicted by genetic factors (i.e. APOE4) and greater WML volume. Alzheimer’s disease pathology is also predicted by positive family history of dementia, lower BMI and lower MMSE score, and combined with WML volume, these improved the discriminative accuracy of a ROC model predicting Alzheimer’s disease versus normal AD biomarkers by 7%, compared to a model including age, sex and APOE e4. Models that included previously validated composite risk scores for dementia, rather than cumulative risk factors, showed no improvement. Greater age was the only predictor of non-AD pathology among cognitively healthy adults, and higher WML volume was the only significant predictor of combined AD/non-AD pathologic change.

The ATN framework was proposed as an unbiased descriptive approach for defining distinct multidomain biomarker profiles at the individual person level [16]. The ATN framework departed from previous criteria by segregating pathologic tau measures from other markers of neuronal injury, as means of improving the ability to differentiate AD from non-AD pathology. The relevance of the ATN system to preclinical disease was demonstrated by data showing that among cognitively healthy individuals, ATN AD pathology status associates with worse cognition [3]. Our analysis extends these results by demonstrating in the largest reported cognitively healthy ageing group to-date that close to half of all participants can be classified as abnormal through ATN criteria (20.9% Alzheimer’s pathologic change, 6.6% Alzheimer’s disease pathology, 3.1% combined AD and non-AD pathologic change and 13.3% non-AD pathology).

The clear utility of ATN is counterweighed by its reliance on sampling AD biomarkers which is invasive and costly. Therefore, accurate prediction of ATN status through more accessible means is a priority. Our analysis demonstrated that a base model encompassing the best validated constitutional risk factors for AD in a preclinical group (age, sex and APOE4 carriership) performed well (AUC 0.82) in predicting AD pathology, with the model being considerably less accurate for AD pathologic change and non-AD pathology (AUCs 0.63 and 0.63, respectively), and particularly combined AD and non-AD pathologic change (AUC 0.56). A recent study showed similar AUCs (0.83 in Pathfinder and 0.82 in Alzheimer’s Disease Neuroimaging Initiative cohorts, respectively) for predicting amyloid positivity using a model combining age, APOE4 and episodic memory [35]. While this analysis was not able to differentiate between AD pathology (i.e. A+T+N+/−) and AD pathologic change (A+T−N−), the two cohorts had a substantial proportion of MCI and subjective memory impairment participants, which makes it likely that they had significant tau pathology. The predictive power of APOE4 for both amyloid and tau positivity in preclinical disease is underlined by another analysis from our group demonstrating that APOE4 carriership is by far the best factor interacting with age on the timing of rapid accumulation of amyloid among cognitively healthy individuals [28].

Our Alzheimer’s disease pathology base model (age, sex and APOE4) were improved by the addition of BMI and WML volume combined with family history and MMSE. We found that it was low BMI that drove the improvement, which replicates established data on a bidirectional relationship between BMI and dementia risk: higher BMI in mid-age and lower BMI in the period immediately before diagnosis [23, 36]. A recent report among initially cognitively healthy women confirmed that low BMI predicts dementia conversion within 5 years but not for longer periods [10]. The lowering of BMI in the years proximal to dementia onset has been attributed to disease prodrome associated changes in eating behaviours, activity and metabolism. There was a small degree of improvement to the AD pathology models by WML volume which is in line with evidence that cerebrovascular pathology co-exists with AD pathology [45]. White matter changes have a close relationship with cardiovascular risk factors such as hypertension [51] and diabetes [48] and are predictive of dementia [2]. Deficits in the cerebral microcirculation are thought to play a role in the propagation of AD pathology through restriction of blood flow, neuroinflammation and amyloid clearance mechanisms [8, 22]. The ATN position paper thus highlighted the potential inclusion of a vascular (V) factor in future revisions of the framework [17]. The currently reported marginal association of WML to AD pathology but not other ATN groups in pre-syndromal individuals warrants further investigation of WML volume as a potential vascular biomarker in future revisions of the ATN framework.

In contrast to BMI and WML volume, we did not observe any improvement to the base model by the addition of the best validated composite risk scores for dementia (CAIDE, Framingham cardiovascular and stroke score). The reason for this may lie in the fact that they were either developed (CAIDE) or repurposed (Framingham scores) to estimate the risk for imminent dementia conversion rather than for use in the potentially more heterogeneous and less well-defined preclinical dementia phenotype. Also, the data reduction inherent in risk scores likely reduces their power to discriminate between pathological and non-pathological preclinical states. Our data suggest that continuous cardiovascular risk factors, as well as proxies of subclinical cerebrovascular disease such as WML volume, may be more relevant in this disease stage.

The analysis was also notable for the relatively poor performance of the prediction models for non-AD pathology. This result is unsurprising given the known low prevalence of APOE4 genotype among individuals with suspected non-Alzheimer disease pathology (SNAP) [26, 32]. SNAP is a biomarker-defined condition (amyloid negative, but evidence for neurodegeneration and/or increased tau protein) affecting approximately 23% of cognitively normal adults aged 65 or above [18]. SNAP is heterogenous in terms of progression as well as clinical phenotype (e.g. cognitive profile) that make its distinction challenging and our current analysis reinforces this by showing the low predictive value of risk factors typically associated with AD risk.

In future, ATN prediction is likely to be improved by biomarkers that are more phase-specific to the preclinical disease stage. Specifically, recent improvement to plasma assay sensitivity has allowed the detection of amyloid: amyloid (Abeta 42/40), tau (ptau-181) and neurodegeneration (neurofilament light). Abeta 42/40 has been shown to correlate with amyloid positivity [33] while increases levels of NfL are evident 10 years prior to estimated disease onset in carriers of autosomally dominant mutations for early-onset AD [50]. Most recently, p-tau181 was shown to correlate strongly with both amyloid and tau PET positivity, tau Braak staging [19] as well as to discriminate AD from other causes of dementia, e.g. fronto-temporal dementia [49]. Others have already demonstrated improved amyloid positivity prediction through the addition of Abeta 42/40 but not NfL to age, APOE4 and cognition models [35]. Further gains may be possible through the rapidly developing digital technologies which offer previously unattainable data granularity in terms of cognitive and functional trajectory, via passive (e.g. interaction with digital technology, navigational skills through GPS) and active (e.g. smartphone cognitive testing) data collection [4, 27, 29].

### Limitations

Previous validation of the CAIDE score was based upon a sample with vascular measures at midlife and 20-year follow-up of AD pathological risk, and such distal relationships offer some evidence for the direction of association. Apart from testing for confounders with the inclusion of select covariates, we therefore cannot comment on the direction of association in the current study. A further limitation is that whereas the CAIDE score was validated according to vascular risk scores at on average 50 years of age (and Framingham scores at age 55 years), our sample was 65 years on average. We know that certain CVD risk factors, i.e. BMI and blood pressure, change in their association with dementia risk and/or cognitive impairment with age. Therefore, we may not have expected to see the same set of vascular risk factors predicting AD pathology than were observed in the somewhat younger Finnish and British Whitehall II samples.

We were limited to looking at the contemporaneous associations between vascular risk factors and ATN biomarkers, and a longitudinal analysis of change in these biomarkers would help to illuminate the present study’s findings.

Finally, healthy volunteer cohort studies are limited through a self-selection bias which may lead to individuals unrepresentative of the general population being more likely to volunteer. While this is an important limitation, this initial analysis allows future work to validate the ATN prediction models in larger cohorts, e.g. Dementias Platform UK Clinical Studies Register (www.greatmindsfordementia.org), Brain Health Registry which may be less prone to this bias due to the virtue of their larger size (over 50k participants) and lower bar for participation (online-only data collection) relative to an observational study such as EPAD.

## Conclusions

Despite their ease of use, brain health composite risk scores did not offer an advantage in the detection of either AD or non-AD preclinical pathology relative to a prediction model consisting of age, sex and APOE4 genotype in our sample of older, healthy adults. Further advances to disease and phase-specific ATN prediction may be possible through novel plasma biomarkers and digital technologies.

## Data Availability

The dataset analysed (EPAD LCS V1500; doi:10.34688/epadlcs_v500.1_20.04.29) during the current study is freely available through the EPAD LCS repository (http://ep-ad.org/erap/).
